# Use of a 1.0 Tesla open scanner for evaluation of pediatric and congenital heart disease: a retrospective cohort study

**DOI:** 10.1186/s12968-015-0144-y

**Published:** 2015-05-25

**Authors:** Jimmy C Lu, James C Nielsen, Layne Morowitz, Muzammil Musani, Maryam Ghadimi Mahani, Prachi P. Agarwal, El-Sayed H. Ibrahim, Adam L. Dorfman

**Affiliations:** Department of Pediatrics and Communicable Diseases, Division of Pediatric Cardiology, University of Michigan, Ann Arbor, MI USA; Department of Radiology, Section of Pediatric Radiology, University of Michigan, Ann Arbor, MI USA; Departments of Pediatrics and Radiology, Stony Brook University, Stony Brook, NY USA; Department of Medicine, Division of Cardiology, Stony Brook University, Stony Brook, NY USA; Department of Radiology, Division of Cardiothoracic Radiology, University of Michigan, Ann Arbor, MI USA

**Keywords:** Open scanner, Congenital heart disease, 1.0 Tesla

## Abstract

**Background:**

Open cardiovascular magnetic resonance (CMR) scanners offer the potential for imaging patients with claustrophobia or large body size, but at a lower 1.0 Tesla magnetic field. This study aimed to evaluate the efficacy of open CMR for evaluation of pediatric and congenital heart disease.

**Methods:**

This retrospective, cross-sectional study included all patients ≤18 years old or with congenital heart disease who underwent CMR on an open 1.0 Tesla scanner at two centers from 2012–2014. Indications for CMR and clinical questions were extracted from the medical record. Studies were qualitatively graded for image quality and diagnostic utility. In a subset of 25 patients, signal-to-noise (SNR) and contrast-to-noise (CNR) ratios were compared to size- and diagnosis-matched patients with CMR on a 1.5 Tesla scanner.

**Results:**

A total of 65 patients (median 17.3 years old, 60% male) were included. Congenital heart disease was present in 32 (50%), with tetralogy of Fallot and bicuspid aortic valve the most common diagnoses. Open CMR was used due to scheduling/equipment issues in 51 (80%), claustrophobia in 7 (11%), and patient size in 3 (5%); 4 patients with claustrophobia had failed CMR on a different scanner, but completed the study on open CMR without sedation. All patients had good or excellent image quality on black blood, phase contrast, magnetic resonance angiography, and late gadolinium enhancement imaging. There was below average image quality in 3/63 (5%) patients with cine images, and 4/15 (27%) patients with coronary artery imaging. SNR and CNR were decreased in cine and magnetic resonance angiography images compared to 1.5 Tesla. The clinical question was answered adequately in all but 2 patients; 1 patient with a Fontan had artifact from an embolization coil limiting RV volume analysis, and in 1 patient the right coronary artery origin was not well seen.

**Conclusions:**

Open 1.0 Tesla scanners can effectively evaluate pediatric and congenital heart disease, including patients with claustrophobia and larger body size. Despite minor artifacts and differences in SNR and CNR, the majority of clinical questions can be answered adequately, with some limitations with coronary artery imaging. Further evaluation is necessary to optimize protocols and image quality.

## Background

Cardiovascular magnetic resonance (CMR) is an indispensible tool for evaluation of cardiac anatomy and physiology in pediatric and congenital heart disease. CMR is particularly useful for adults with congenital heart disease, in whom acoustic windows often limit the utility of echocardiography [1, 2]. However, this population may be at increased risk for obesity [3, 4], which can limit use of CMR. Short bore 1.5 Tesla scanners, the primary tool in this population, or 3.0 Tesla scanners can be limited by patient size and difficulty with claustrophobia, as patients must lie still within a circumferential bore. "Open" scanners allow more room to the sides of the patient, and may be more tolerable for patients with claustrophobia or larger body size [5]. At a lower magnetic field of 1.0 Tesla, there are also theoretical advantages, e.g. lower specific absorption rate (SAR) and less B1 shading artifact. However, there is a paucity of data regarding application of 1.0 Tesla scanners in pediatric and congenital heart disease [6, 7], and it is unclear whether the lower magnetic field and signal to noise ratio is adequate to evaluate pediatric patients with smaller body size, or adult congenital heart disease patients, with more complicated cardiac anatomy. This retrospective study aimed to evaluate the efficacy of open scanners in this population.

## Methods

### Patient selection

This multicenter retrospective cohort study included all patients who underwent CMR for evaluation of pediatric or congenital heart disease on an open scanner at Stony Brook University or the University of Michigan C.S. Mott Children’s Hospital, between 2/8/2012 and 7/24/2014. This study was approved by the Institutional Review Boards at both centers, and requirement for informed consent in this retrospective study was waived. Patients of any age with congenital heart disease were included; patients without congenital heart disease were included if they were 18 years or younger at the time of CMR. Patients were excluded if digital images were not available for review. Medical records were reviewed for patient demographics, indication for the study, and reason for performing the study on the open scanner.

For quantitative comparison of signal-to-noise ratio (SNR) and contrast-to-noise ratio (CNR), a subset of 25 CMR studies were matched 1:1 by patient body surface area and diagnosis with CMR studies performed on a 1.5 Tesla scanner at the same institution. It should be noted that SNR and CNR depend on a number of imaging parameters, including voxel size, acceleration factor, readout bandwidth, and number of averages. Therefore, imaging parameters on both scanners are summarized in Table 1.

**Table 1:** Typical imaging parameters at 1.0 Tesla and 1.5 Tesla

	1.0 Tesla	1.5 Tesla
**Cine SSFP**		
TR (ms)/TE (ms)	4.1-4.4/2.0-2.2	3.1-3.3/1.6
Flip angle (°)	55-60	60
Receive bandwidth (Hz/pixel)	578 or 1014	954
In-plane spatial resolution (mm)	1.9-2.0	1.7-1.9
Slice thickness (mm)	8	8
Phases per cardiac cycle	24-30	30
SENSE	1	2
SAR*	<74-92%	<95%
**Black blood**		
In-plane spatial resolution (mm)	1.6-1.7	1.2-1.5
Slice thickness (mm)	5-7	4-5
Number of signals averaged	1-2	1
SENSE	1	2
SAR (for proton density)	<34%	<15%
**Phase contrast**		
TR (ms)/TE (ms)	6.5-6.9/4.1-4.7	3.1-3.3/1.6
Flip angle (°)	12-25	12
Receive bandwidth (Hz/pixel)	318-364	717
In-plane spatial resolution (mm)	2.0-2.2	1.6-1.8
Slice thickness (mm)	6	6
Phases per cardiac cycle	20-40	40
Number of signals averaged	2-3	3
SENSE	1	2
SAR	<2%	<2%
**Magnetic resonance angiogram**		
TR (ms)/TE (ms)	4.0/1.5	4.8/1.5
Flip angle (°)	40	40
Receive bandwidth (Hz/pixel)	482 or 789	249
In-plane spatial resolution (mm)	1.8-2.2	1.7-1.8
Slice thickness (mm)	3.0-3.4	2.6-2.8
SENSE	1	1.5 AP, 1.5 LR
SAR	<82-92%	<51%
**3-dimensional SSFP**		
TR (ms)/TE (ms)	5.1-5.5/2.6-2.8	4.3/2.2
Flip angle (°)	90	90
Receive bandwidth (Hz/pixel)	578	618
Isotropic voxel size (mm)	1.9-2.6	1.6
SENSE	1	1.5 AP, 1.5 LR
SAR	<62%	<36%
**LGE**		
TR (ms)	6.0	6.1
TE (ms)	3.0	3.0
Flip angle (°)	25	25
Receive bandwidth (Hz/pixel)	299 or 495	232
In-plane spatial resolution (mm)	2.0-2.2	1.7-1.8
Slice thickness (mm)	8	8
SENSE	1	1
SAR	<10%	<8%
**3-dimensional LGE**		
TR (ms)/TE (ms)	5.3/2.6	Not performed
Flip angle (°)	25	
Receive bandwidth (Hz/pixel)	299	
In-plane spatial resolution (mm)	1.9	
Slice thickness (mm)	5	
SAR	<17%	

SSFP, balanced steady-state free precession; TR, repetition time; TE echo time; SENSE, sensitivity encoding; SAR, systemic absorption rate; LGE, late gadolinium enhancement

*SAR measurements are based on level 0 (normal operating mode) with whole body SAR < 2 W/kg

### CMR imaging

CMR was performed with a Panorama High Field Open 1.0 Tesla scanner (Philips, Best, The Netherlands), using a solenoid body coil (medium, large or extra large as appropriate for patient size). Gradient amplitude is 28 mT/m; maximum slew rate is 120 mT/m/ms. Sensitivity encoding (SENSE) was not used for image acquisition, i.e. SENSE acceleration factor = 1, to maintain adequate signal-to-noise ratio (SNR). Typical imaging parameters are presented in Table 1, with some minor differences by center. Cine imaging was performed with a breathhold, electrocardiographic gated, segmented k-space balanced steady-state with free precession (SSFP) sequence. Black blood imaging was performed with a breathhold, electrocardiographic gated, double inversion recovery turbo spin echo sequence. Imaging parameters varied by weighting of the sequence. Phase contrast imaging was performed free-breathing. Magnetic resonance angiogram (MRA) was performed with a T1-weighted fast field echo sequence, after injection of 0.2 mmol/kg of gadoteridol (ProHance, Bracco, Monroe Township, New Jersey) for studies performed at the University of Michigan, or gadopentetate dimeglumine (Magnevist, Bayer, Leverkusen, Germany) for studies performed at Stony Brook University. Two post-contrast dynamics were performed to highlight the anatomy of interest (e.g. pulmonary arteries or aorta) as well as the venous phase. Coronary artery imaging was performed with a respiratory navigator gated, electrocardiographic gated, three-dimensional SSFP sequence. Late gadolinium enhancement (LGE) was performed 12–15 minutes after contrast injection, using a breathhold, electrocardiographic gated, phase-sensitive inversion recovery sequence. In 12 patients at the University of Michigan, LGE imaging was performed with a three-dimensional, respiratory navigator gated, electrocardiographic gated, phase-sensitive inversion recovery sequence, to improve in-plane spatial resolution while maintaining adequate SNR.

CMR at 1.5 Tesla was performed on a commercially available scanner (Ingenia, Philips, Best, The Netherlands), using a phased-array body surface coil. Gradient amplitude is 33 mT/m; maximum slew rate is 200 mT/m/ms. Typical imaging parameters are presented in Table 1. It should be noted that TR was longer on the 1.0 Tesla scanner than the 1.5 Tesla scanner due to the lower gradient performance characteristics (maximum gradient strength and slew rate) of the 1.0 Tesla scanner. Phase contrast imaging was performed free-breathing. MRA was performed with two post-contrast dynamics, which was sufficient to highlight the anatomy of interest, e.g. pulmonary arteries or aorta, as well as the venous phase of the contrast uptake. LGE was performed using a breathhold, electrocardiographic gated, phase-sensitive inversion recovery sequence.

Scan time for both scanners was measured from the start of the first imaging sequence through the final imaging sequence. Thus, this included patient rest time between breath-holds, and potential need to pause during imaging if there was any patient discomfort.

### Image analysis

**Fig. 1:**
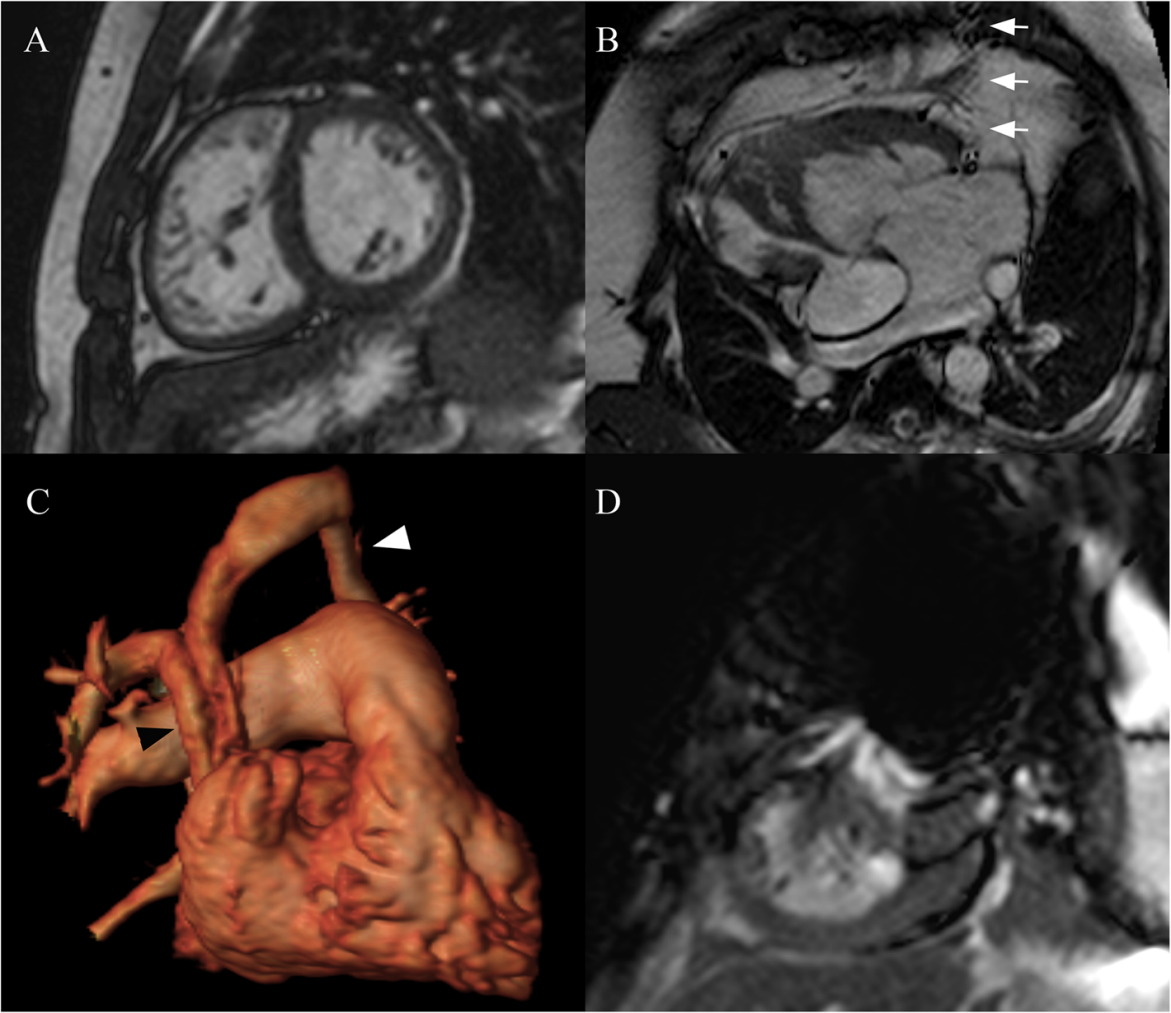
Images were scored on a 4-point scale for image quality and diagnostic utility. **(a)** SSFP imaging in short-axis in a 26 year-old patient with tetralogy of Fallot and complete atrioventricular septal defect status post repair: No artifacts and good endocardial definition, scored 4 for both quality and diagnostic utility. **(b)** SSFP imaging in the four-chamber plane in a 32 year-old patient with dextrocardia and congenitally corrected transposition of the great arteries, who previously failed CMR on a 1.5 Tesla scanner: Flow-related artifact (arrows), which did not affect interpretation of ventricular size or function, scored 3 for quality, but 4 for diagnostic utility. **(c)** Volume-rendered reconstruction of gadolinium-enhanced MRA in a 57 year-old patient with pulmonary hypertension and reported history of atrial septal defect repair in a foreign country. The left upper pulmonary vein (white arrowhead) drains into the left innominate vein; the right upper and middle veins drain into a baffle within the superior vena cava (black arrowhead) to the left atrium. Scored 4 for both quality and diagnostic utility. **(d)** SSFP imaging in short axis in a 16 year-old patient with unbalanced atrioventricular septal defect status post Fontan: Significant coil artifact, obscuring portions of the heart, scored 2 for both quality and diagnostic utility (for right ventricular size and function)

All images were evaluated by a single experienced observer. For each sequence type, image quality was scored qualitatively, taking into consideration resolution, blurring effects, low SNR, delineation of structures, and artifacts, according to a 4-point scale (4 – excellent with no artifacts; 3 – good with minor artifacts; 2 – below average, with significant artifacts affecting interpretation; 1 – poor, nondiagnostic) [8]. Imaging examples are presented in Fig. 1.

Clinical questions were extracted from the medical record, and were categorized as: ventricular size/function, pulmonary artery anatomy, regurgitant fraction, LGE, aortic root dimensions, coronary artery anatomy, aortic arch anatomy, ratio of pulmonary to systemic blood flow (Qp:Qs ratio), and pulmonary venous anatomy. A given study could have multiple clinical questions. Studies were scored for diagnostic utility on a 4-point scale, based on the interpreter’s confidence in their ability to answer the specific clinical questions (4 – high confidence of diagnosis; 3 – answers question adequately; 2 – low confidence; 1 – could not answer the clinical question). Ventricular size/function was scored on the ability to define and contour the endocardial border. Anatomy of the pulmonary arteries, coronary arteries, aortic arch, and pulmonary veins was scored on definition of the respective structures, course, and presence/absence of stenosis as appropriate. Flow measurements were scored by internal consistency of data. LGE images were scored by ability to determine presence or absence of LGE, and extent if present.

Due to the qualitative nature of grading image quality and diagnostic utility, a 20% subset of the cohort was randomly selected to evaluate reproducibility. A second reader, blinded to the scores of the first reader, re-evaluated the images to evaluate interobserver agreement. The initial reader, blinded to initial scores, also re-evaluated the images to evaluate intraobserver agreement.

**Fig. 2:**
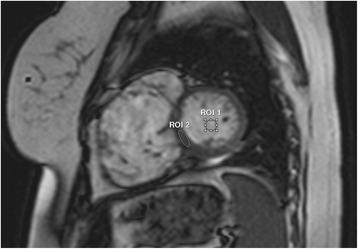
Regions of interest for contrast to noise measurement. For cine images, an ROI of ~1.0 cm^2^ was drawn in the blood pool and in the septum. Only ROI1 (the blood pool) was used for signal to noise ratio measurement

SNR and CNR were calculated for cine SSFP, black blood, MRA, 3-dimensional SSFP, and LGE images. A region of interest (ROI) of approximately 1.0 cm^2^ was drawn in two locations (Fig. 2). For cine SSFP and LGE images, a midventricular short-axis slice was used, and ROIs were drawn in the blood pool and in the interventricular septum. For black blood and 3-dimensional SSFP images, ROIs were drawn in the blood pool and in any adjacent myocardium. For the MRA, the first post-contrast dynamic was used, and ROIs were drawn in the aorta or main pulmonary artery (depending on timing of the contrast) and in the lung field. SNR was defined as the mean signal intensity in the blood pool (for cine SSFP, MRA, 3-dimensional SSFP and LGE images) or myocardium (for black blood images) divided by the standard deviation of signal intensity in that ROI [9]. CNR was defined as the difference in signal intensities of the two ROIs, divided by the average of the standard deviations of the two ROIs [9].

### Statistical analysis

Data are presented as median (interquartile range [IQR]), mean ± standard deviation, or number (percent), as appropriate. SNR, CNR, and scan time on 1.0 Tesla and 1.5 Tesla studies were compared for each sequence using t-test. Age and body surface area among patients on 1.0 Tesla and 1.5 Tesla patients were not normally distributed, and were compared using Wilcoxon matched-pairs signed rank test. A p-value < 0.05 was considered statistically significant. Intraobserver and interobserver agreement was calculated using weighted kappa, to take into consideration closeness of agreement.

## Results

A total of 65 patients were included, with demographic data presented in Table 2. The majority of the patients were adolescents and adults, with 6 patients under the age of 10, and the youngest 3.5 years old. Patients were referred to the open scanner primarily due to scheduling reasons, although 10 (15%) were referred for claustrophobia or patient size. Among patients referred for claustrophobia or body size, 4 had failed a previous attempt on a 1.5 Tesla scanner; all 4 patients successfully completed the study on the open scanner, without sedation. Of the remaining 6 patients, 4 were referred for first CMR on the open scanner; 2 had previously had a successful scan on a standard scanner. Only 2 patients in this cohort required sedation, due to age or developmental delay. Apart from sternal wires (20/65 patients, 31%), implanted metal objects were rare (1 with an embolization coil, 1 with a pulmonary artery stent, 1 with Harrington rods). Mean scan time was 54.1 ± 22.4 minutes. In the matched cohort of 1.0 Tesla and 1.5 Tesla patients, there was no significant difference in scan time (58.9 ± 20.9 vs. 51.7 ± 12.7 min, p = 0.15).

**Table 2:** Demographic data (N = 65)

Age (years)	17.3 (14.7-20.9)
Male gender	39 (60%)
Height (cm)	166 (154–175)
Weight (kg)	71.0 (60.5-87.7)
Body surface area (m^2^)	1.83 (1.67-2.04)
Diagnosis of congenital heart disease	33 (51%)
Tetralogy of Fallot s/p repair	8
Bicuspid aortic valve	6
Aortic stenosis	4
Coarctation s/p repair	2
Ebstein anomaly	2
Partial anomalous pulmonary venous connections	2
Pulmonary stenosis	2
Other	7
Indications for CMR	
Left ventricular size/function	53 (82%)
Right ventricular size/function	26 (40%)
Regurgitant fraction	23 (35%)
Pulmonary artery anatomy	16 (25%)
Aortic root dimensions	15 (23%)
Late gadolinium enhancement	15 (23%)
Coronary artery anatomy	12 (18%)
Aortic arch anatomy	10 (15%)
Qp:Qs ratio	7 (11%)
Pulmonary venous anatomy	4 (6%)
Reason for open scanner	
Scheduling/equipment issue	52 (80%)
Claustrophobia	7 (11%)
Patient size	3 (5%)
Unspecified	3 (5%)

Data given as median (IQR) or number (percent). Qp:Qs, ratio of pulmonary to systemic blood flow

### Image quality

**Fig. 3:**
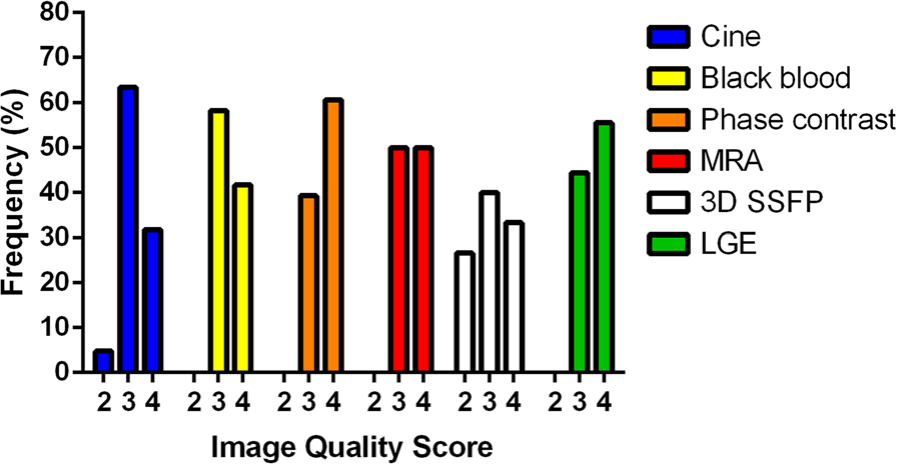
Histogram of image quality score, by imaging sequence. MRA, magnetic resonance angiogram. 3D SSFP, three-dimensional steady state free precession. LGE, late gadolinium enhancement

All patients had good or excellent image quality on black blood, phase contrast, MRA, and LGE imaging (Fig. 3). There was below average image quality in 3/63 (5%) patients with cine images, and 4/15 (27%) patients with coronary artery imaging; all other sequences had at least good image quality in all patients. No sequences were rated as poor/nondiagnostic. Intraobserver agreement for grading image quality was good, with weighted kappa of 0.73. Interobserver agreement was fair, with weighted kappa of 0.32. The majority of disagreement was in differentiating between good and excellent image quality; the raters had 85% agreement in differentiating image quality 2 from values of 3–4.

**Fig. 4:**
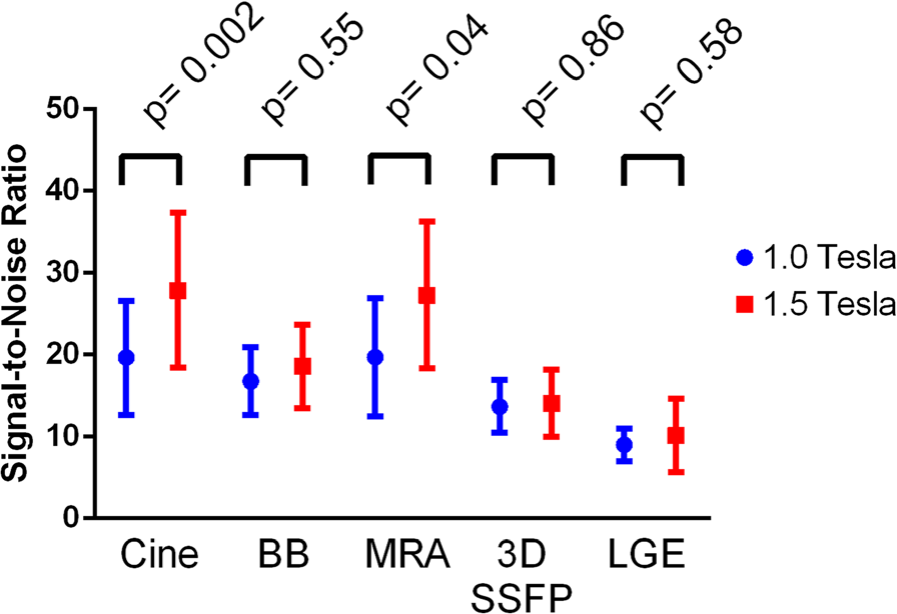
Signal-to-noise ratio by sequence on 1.0 Tesla open or standard 1.5 Tesla scanner. BB, black blood; MRA, magnetic resonance angiogram; 3D SSFP, 3-dimensional steady state free precession; LGE, late gadolinium enhancement

**Fig. 5:**
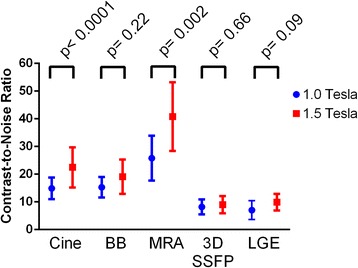
Contrast-to-noise ratio by sequence on 1.0 Tesla open or standard 1.5 Tesla scanner. BB, black blood; MRA, magnetic resonance angiogram; 3D SSFP, 3-dimensional steady state free precession; LGE, late gadolinium enhancement

In the subset of 25 patients for SNR and CNR analysis, there was no difference between patients on the 1.0 Tesla and 1.5 Tesla scanners in age (median 20.6, IQR 15.4-34.9, vs. 22.4, IQR 16.0-22.2, p = 0.60) or body surface area (1.83, IQR 1.62-2.09, vs. 1.84, IQR 1.66-2.07, p = 0.62). Both SNR (Fig. 4) and CNR (Fig. 5) were decreased on the open scanner for cine SSFP and MRA images, but similar to the 1.5 Tesla scanner for black blood, 3-dimensional SSFP, and LGE images. Use of 3-dimensional LGE imaging markedly increased SNR (26.5 ± 12.3 vs. 9.0 ± 0.8, p = 0.004) and CNR (19.2 ± 5.9 vs. 7.0 ± 1.4, p = 0.0003) compared to 2-dimensional LGE imaging at 1.0 Tesla.

### Diagnostic utility

**Fig. 6:**
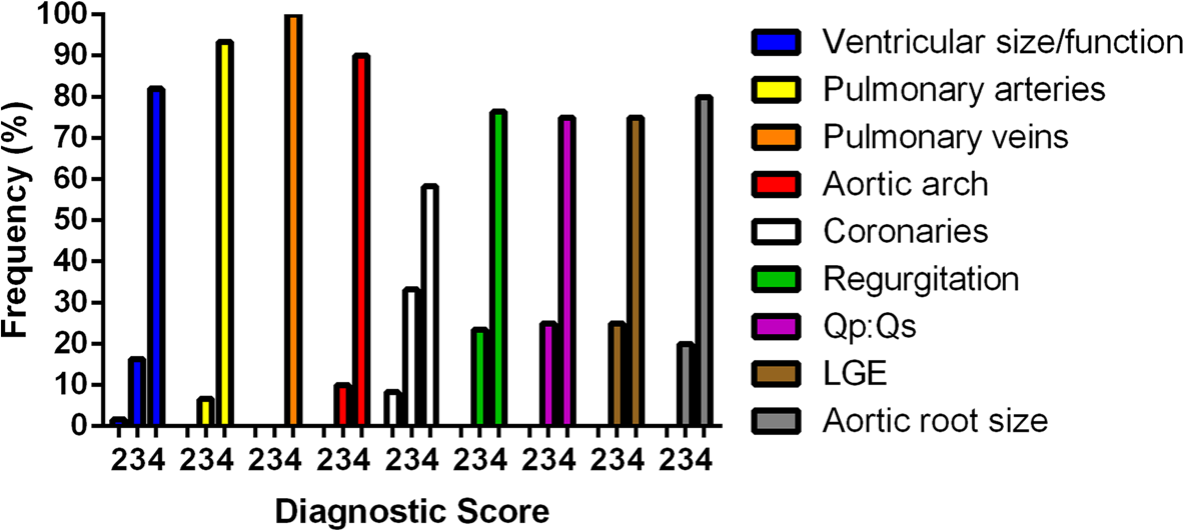
Histogram of diagnostic utility, by clinical question. Qp:Qs, ratio of pulmonary to systemic blood flow. LGE, late gadolinium enhancement

The clinical question was answered adequately (score 3+) in all but 2 patients (Fig. 6). In 1 patient status post Fontan procedure, susceptibility artifact due to an embolization coil limited evaluation of right ventricular volume. In 1 patient, the right coronary artery course appeared normal, although the origin was suboptimally visualized; the left coronary artery was normal. No studies were scored as nondiagnostic, and no patients required callback for re-evaluation on a different scanner. Intraobserver and interobserver agreement for grading diagnostic utility was good, with weighted kappa 0.85 and 0.61, respectively. Again, the vast majority of disagreement was differentiating whether the clinical question was answered adequately or confidently; there was 97% agreement in differentiating score 2 from score 3–4.

## Discussion

Open 1.0 Tesla scanners can be effective for evaluation of selected groups of patients with pediatric and congenital heart disease. Despite minor artifacts and decreased SNR and CNR, the majority of clinical questions can be answered adequately. To our knowledge, this is the first systematic report of CMR at 1.0 Tesla in the pediatric and congenital heart disease population.

The primary advantage of open scanners is the ability to increase the population eligible for CMR, including patients with larger body size or claustrophobia. A prior study demonstrated decreased anxiety in claustrophobic patients undergoing open CMR [5], although a randomized controlled trial demonstrated a significant persistent rate of claustrophobic events in the open scanner [10]. These studies included primarily patients with magnetic resonance imaging of other body parts; patients undergoing CMR, who must have the torso within the bore, could potentially be at higher risk for failure of CMR in a closed bore scanner. Although limited to a small subset of this cohort, the current results are promising, as all 4 patients who had failed a prior scan on a closed bore scanner completed the study on the open scanner without sedation. Prospective identification of patients who may benefit from the open scanner could prevent lost scanner time or need to reschedule.

In this cohort, the majority of patients were referred to the open scanner due to scheduling or equipment issues, suggesting the primary practical advantage may be availability of scanner time. However, the diagnostic success of open CMR studies in this cohort confirms that selected patients in this population can appropriately take advantage of this scanner. Further work is needed to identify additional populations that can appropriately take advantage of open scanner availability.

Due to the lower magnetic field, signal to noise ratio suffers, and modifications to existing protocols are necessary to optimize image quality. De Bucourt and colleagues reported decreased SNR and CNR in extremely obese patients with an open 1.0 Tesla scanner [11]. However, this study used the integrated body coil, rather than the standard body coil, which was used for regular weight control patients. All patients in the current study used the standard body coil, which may partially explain more similar SNR and CNR in some sequences to studies at 1.5 Tesla. Furthermore, techniques to maximize signal, such as increased voxel size, avoidance of parallel imaging [12], and multiple signal averaging may partially offset differences due to magnetic field strength, at the cost of scan time and spatial resolution. In this cohort, studies on the 1.0 Tesla scanner had lower spatial resolution with more frequent usage of multiple signal averaging, without parallel imaging. By adjusting these parameters, SNR and CNR were actually similar among 1.0 Tesla and 1.5 Tesla scanners for black blood, 3-dimensional SSFP, and LGE imaging, but remained decreased for cine SSFP images and MRA. SNR and CNR could be further improved for LGE imaging with a 3-dimensional sequence, which is consistent with prior data at 1.5 Tesla [13]. Further evaluation is necessary for optimization of image quality, to minimize potential for diagnostic error. Furthermore, more technical and quantitative analysis may be necessary particularly for phase contrast data. Longer echo time has been shown to increase risk for error in flow measurements, due to intravoxel dephasing, particularly with stenotic jets [14-16]. This retrospective study did not allow for direct comparison of flow measurements in the same patients at 1.0 Tesla and 1.5 Tesla.

Despite some differences in image quality, the diagnostic score best reflects the clinical potential of the open scanner. Pertinent clinical findings were still detectable, particularly when the focus was ventricular size and systolic function. However, further optimization remains necessary, particularly for coronary artery imaging, which was the most limited of the indications in this study, or when evaluating small vessels. This cohort included few small children; further study is necessary, but patient selection based on clinical indication may be necessary.

This study has several limitations. Retrospective chart review is limited by documentation for determining reasons for the study or reasons for using the open scanner. However, only 3 patients did not have an identified reason for the open scanner. The retrospective nature of this study also prevented standardization of the imaging protocols, particularly between centers; further prospective study is necessary to optimize imaging parameters at 1.0 Tesla. This cohort consisted primarily of adolescents and adults, which may reflect selection bias. Although extrapolation to younger patients may not be appropriate, this does highlight that this population may benefit from use of the open scanner. This cohort cannot address whether need for sedation in smaller children can be lessened with the open scanner. The small sample size limits power to detect small differences in SNR and CNR, and verification in a larger cohort is necessary.

## Conclusions

Open 1.0 Tesla scanners can successfully evaluate pediatric and congenital heart disease in selected patients. Despite some differences in image quality, SNR and CNR, the vast majority of studies were adequate to answer the clinical questions. Further work is necessary to optimize protocols for these indications, and to better define patient characteristics appropriate to this scanner. Use of the open scanner in this population could increase the potential cohort who can tolerate CMR without sedation or discomfort.
